# Gravitational Hamiltonian Systems and the Retarded Gravity Inequality

**DOI:** 10.3390/e26110986

**Published:** 2024-11-17

**Authors:** Asher Yahalom

**Affiliations:** 1 Department of Electrical & Electronic Engineering, Faculty of Engineering, Ariel University, Ariel 40700, Israel; asya@ariel.ac.il; Tel.: +972-54-7740294; 2 Center for Astrophysics, Geophysics, and Space Sciences (AGASS), Ariel University, Ariel 40700, Israel

**Keywords:** gravity, general relativity, dark matter

## Abstract

Gravity and electromagnetic interactions are the only fundamental physical interactions (outside the nuclear domain). In this work, we shall concentrate on Hamiltonians containing gravitational interaction, which according to general relativity must be retarded. In recent years, retarded gravity has explained many of the mysteries surrounding the “missing mass” related to galactic rotation curves, the Tully–Fisher relations, and gravitational lensing phenomena. Indeed, a recent paper analyzing 143 galaxies has demonstrated that retarded gravity will suffice to explain galaxies’ rotation curves without the need to postulate dark matter for multiple types of galaxies. Moreover, it also demystified the “missing mass” related to galactic clusters and elliptic galaxies in which excess matter was derived through the virial theorem. Here, we give a mathematical criterion that specifies the cases in which retardation is important for gravity (and when it is not). The criterion takes the form of an inequality.

## 1. Introduction

From a practical standpoint, general relativity (GR) has been validated by numerous observations spanning various fields. However, its current status presents challenges. While supported by substantial observational evidence, GR faces significant hurdles. Its verifications in cosmology and astrophysics are under scrutiny, primarily because it relies on unproven concepts like dark matter and energy to explain phenomena on a large scale, such as galaxies and the universe. Often, these unconfirmed elements are employed while simultaneously overlooking a crucial aspect of GR, retardation, which contradicts Newtonian principles of action at a distance. This discrepancy may be connected to the problems we have understanding of gravitational interactions on cosmic scales.

The mystery surrounding dark matter has long been a topic of discussion within the astronomical community, dating back to the 1930s, and possibly even earlier in the 1920s, when it was referred to as the “question of missing mass”. Over time, this enigma has only grown more prominent, particularly as the need for dark matter (and the tendency to overlook retardation) has increased on larger scales under examination. Despite extensive and costly efforts, including a forty-year search conducted underground and using accelerators, dark matter’s existence remains unproven. Recent years have only added to the challenge, as the Large Hadron Collider’s failure to detect any super-symmetric particles—a favored form of dark matter among astroparticle physicists—poses further complications. These particles are not only crucial for understanding dark matter but also play a pivotal role in string theory, which is anticipated to provide insights into the quantization of gravity.

As far back as 1933, Zwicky observed anomalies in the velocities of galaxies inside the Coma Cluster that exceeded predictions based on Newtonian theory. He calculated [1,2] that the required amount of matter to explain these velocities could be about 400 times greater than that of visible matter, although later adjustments mitigated this discrepancy to some extent. Had Zwicky utilized the concept of retarded gravity in his calculations, this issue might have been resolved without significant complication [3]. In 1959, Volders [4] noted similar discrepancies on a smaller scale within the outer parts of the nearby spiral galaxy M33, where velocities did not follow the expected $\frac{1}{\sqrt{r}}$ pattern. This observation was corroborated in subsequent years by Rubin and Ford [5,6,7], who demonstrated that velocities at the outer rim of many spiral galaxies either plateaued or continued to increase, each at a different velocity. Previous studies have indicated that such velocity patterns can be directly inferred from general relativity (GR) if retardation effects are considered [8,9,10,11]. The mechanism underlying retardation is intricately linked to the dynamics of the matter density within galaxies, specifically to the second derivative of density. Changes in density may result from various factors, including gas depletion in surrounding intergalactic gas [10] or dynamic processes such as star formation and supernova explosions [8,9]. These processes can be characterized by three different typical length scales: the density gradient length, velocity field gradient length, and dynamical length scale. The importance of retardation is determined by the shortest among these length scales [12].

The famous Tully–Fisher relation [13], which links the baryonic mass of a galaxy to the fourth power of its rotational velocity at the outer rim, can also be derived from the principles of retarded gravity [14]. It was shown that the effects of retarded gravity extend beyond just slowly moving particles and also apply to photons. While there may be some differences in the mathematical analysis for each case, it is ultimately concluded that the observed “dark mass” inferred from galactic rotation curves must be identical in both the lensing and rotation curves scenarios [12].

While the prevailing notion of dark matter remains prominent, the current circumstances warrant consideration of the case that this prevailing paradigm may need to be reevaluated. Several challenges cast doubt on this common idea:

Firstly, in order to align with observed phenomena and structure formation simulations, a set of properties has been assigned to dark matter (DM) [2]. However, despite being over 50 years since its inception, dark matter has yet to be directly observed, nor have any known particles been identified that match its purported properties.

Secondly, simulations involving dark matter often encounter what is known as the core-cusp problem. The Navarro–Frenk–White (NFW) [15] profile, derived from Cold Dark Matter (CDM) simulations and commonly used to fit rotation curves, is a prime example. However, this profile faces challenges, particularly when applied to Low-Surface-Brightness galaxies (LSBs). Derivations made using the NFW profile regarding rotational velocities frequently diverge from actual observations, leading to discrepancies. Specifically, while the NFW profile anticipates a “cuspy” internal region for a dark halo (where density changes rapidly), observations tend to favor a “core-like” behavior (where density remains approximately constant). Efforts to address this issue have often relied on specific and somewhat contrived adjustments, raising doubts about whether these solutions were devised primarily to maintain the current paradigm.

Thirdly, Sancisi’s Law [16] presents a significant and broadly applicable observation. It suggests that changes in the luminosity profile of a galaxy correspond to changes in its rotation curve, and vice versa. This phenomenon applies to various types of dark halos. However, from a dark matter perspective, this relationship is unexpected: the dark halo is typically assumed to be much more massive than the baryonic matter. Consequently, fluctuations in the distribution of baryonic matter should not significantly affect the velocity distribution, contrary to what is observed. This discrepancy is particularly pronounced in LSBs, where the dark halo is believed to dominate at every radius, yet the velocity distribution exhibits fluctuations corresponding to each “baryonic bump”. This suggests that, somehow, the overall velocity distribution is influenced by small fluctuations in baryonic matter.

Hence, the current retarded gravity proposition offers a unique perspective. Unlike alternative theories that propose modifications to general relativity, such as Milgrom’s Modified Newtonian Dynamics (MOND) [17], Mannheim’s Conformal Gravity [18,19], or Moffat’s Modified Gravity (MOG) [20], our approach does not seek to alter the fundamental framework of general relativity. Instead, we adhere strictly to the principle of Occam’s razor, as advocated by both Newton and Einstein. Our objective is to replace the need for dark matter with phenomena inherent within standard general relativity itself (retardation). It’s worth noting that recent research has shed light on the relationship between retardation and MOND [21], demonstrating how criteria for low acceleration MOND can be deduced from retardation theory, and how the MOND interpolation function can approximate retarded gravity effectively.

It is essential to highlight that significant retardation effects are not contingent upon high velocities of matter within galaxies, although higher velocities may enhance these effects. In reality, the majority of galactic constituents, such as stars and gas, move relatively slowly compared with the speed of light. This is indicated by the ratio of the velocity *v* to the speed of light in vacuum *c*, denoted as $\frac{v}{c}$, which is much smaller than 1. For instance, typical velocities within galaxies are around 100 km/s, resulting in a ratio of 0.001 or smaller. This will be discussed in more detail in the sections that follow.

**Figure 1:**
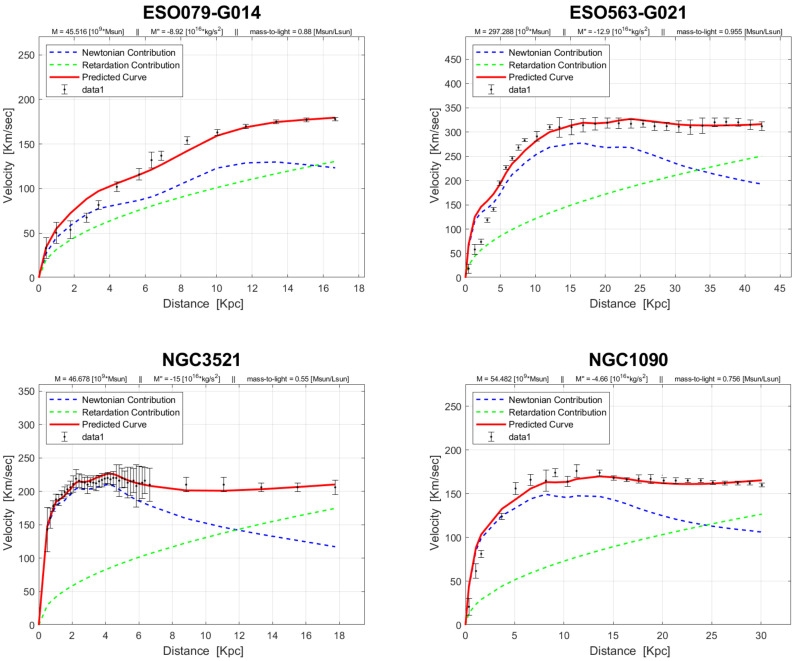
Some rotation curves of ‘Sbc’ type galaxies.

In contrast to the solar system, where retardation effects are considered negligible [22], observations of galaxies’ velocity curves suggest that these effects become significant beyond a certain distance [8,9,10]. Recent research [23] has expanded the empirical basis for the theory of retarded gravity. Building upon previous studies that analyzed eleven galaxies [9], the latest research extends its scope to a larger sample of 143 galaxies sourced from the SPARC Galaxy collection. These galaxies vary in type, size, and luminosity. The analysis indicates that in most cases, an excellent fit to the observed data is achieved without the need to postulate dark matter or modify general relativity (see Figure 1 for some examples).

As we show below, this is not an accident but is rather dictated by general relativity.

## 2. Hamiltonian Systems

Consider a system of *N* particles, each with a displacement ${\vec{x}}_{i}$, mass $m_{i}$, and momentum ${\vec{p}}_{i}$. The index *i* is an integer such that i∈[1−N]. Thus, the dimensions of the phase space of such a system are 6N. The classical dynamics of such a system may be derived from a Hamiltonian *H* and the Hamilton equations:

(1) $$\dot{{\vec{p}}_{i}}=-\frac{\partial H}{\partial {\vec{x}}_{i}},\;\dot{{\vec{x}}_{i}}=\frac{\partial H}{\partial {\vec{p}}_{i}}$$

in which $\dot{f}$ is the temporal derivative of *f*. The Hamiltonian *H* is often partitioned into two parts; kinetic *K* and potential *V*:

(2) $$H\left({\vec{x}}_{1}\ldots {\vec{x}}_{N},{\vec{p}}_{1}\ldots {\vec{p}}_{N}\right)=K\left({\vec{p}}_{1}\ldots {\vec{p}}_{N}\right)+V\left({\vec{x}}_{1}\ldots {\vec{x}}_{N}\right)$$

In which:

(3) $$K=\sum_{i=1}^{N}\frac{{\vec{p}}_{i}^{2}}{2m_{i}},\;V=\sum_{i=1,j>i}^{N}\phi_{ij}\left({\vec{x}}_{i},{\vec{x}}_{j}\right).$$

Now, in nature, there are only four fundamental forces: electromagnetic, gravitational, nuclear strong, and nuclear weak. The last two are only relevant for the nuclear and elementary particle scales. Thus, for classical systems, the only important potentials are electromagnetic and gravitational. In both cases, the two body interaction potentials $\phi_{ij}$ of the type appearing in Equation (3) must be retarded. This is due to the structure of the Maxwell equations in the electromagnetic case and the structure of general relativity for the gravitational case. It is the purpose of this paper to derive criteria in which the retarded potentials may be replaced by an action at a distance potential; this is discussed in the following sections.

## 3. A Discrete Model

Retarded gravity emerges from the weak field approximation of general relativity [10]. In this framework, the metric perturbation $h_{00}$ can be expressed in terms of a retarded potential ϕ as follows [10,12]:

(4) $$\phi =-G\int \frac{\rho ({\vec{x}}^{\prime },t-\frac{R}{c})}{R}d^{3}x^{\prime },\;\phi \equiv \frac{c^{2}}{2}h_{00},\;h_{00}=\frac{2}{c^{2}}\phi$$

where *G* represents the gravitational constant, $\vec{x}$ denotes the location where the potential is calculated, ${\vec{x}}^{\prime }$ signifies the whereabouts of the mass element producing the potential, $\vec{R}\equiv \vec{x}-{\vec{x}}^{\prime },\;R\equiv \left|\vec{R}\right|$, and ρ represents the mass distribution. The characteristic duration $\frac{R}{c}$ for galaxies might span a few tens of thousands of years, but this is small relative to the timescale over which galactic density significantly changes. Therefore, we can express the density using a Taylor series expansion:

(5) $$\rho \left({\vec{x}}^{\prime },t-\frac{R}{c}\right)=\sum_{n=0}^{\infty }\frac{1}{n!}\rho^{\left(n\right)}\left({\vec{x}}^{\prime },t\right){\left(-\frac{R}{c}\right)}^{n},\;\rho^{\left(n\right)}\equiv \frac{\partial^{n}\rho }{\partial t^{n}}.$$

By substituting Equation (5) into Equation (4) and retaining the first three terms, we can derive the following:

(6) $$\phi =-G\int \frac{\rho ({\vec{x}}^{\prime },t)}{R}d^{3}x^{\prime }+\frac{G}{c}\int \rho^{\left(1\right)}\left({\vec{x}}^{\prime },t\right)d^{3}x^{\prime }-\frac{G}{2c^{2}}\int R\rho^{\left(2\right)}\left({\vec{x}}^{\prime },t\right)d^{3}x^{\prime }$$

The initial term in the series is referred to as the Newtonian potential:

(7) $$\phi_{N}=-G\int \frac{\rho ({\vec{x}}^{\prime },t)}{R}d^{3}x^{\prime }$$

The second term does not influence the force acting on subluminal particles since its gradient is zero. As for the third term, it serves as a lower-order amendment to the Newtonian potential.

(8) $$\phi_{r}=-\frac{G}{2c^{2}}\int R\rho^{\left(2\right)}\left({\vec{x}}^{\prime },t\right)d^{3}x^{\prime }$$

The geodesic equation governing the motion of a “slow” test particle in the given space–time metric can be evaluated by utilizing the force per unit mass [10]:

(9) $$\vec{a}\equiv \frac{d\vec{v}}{dt}=-\vec{\nabla }\phi .$$

The total acceleration is thus as follows:

(10) $$\begin{array}{ccc} \vec{a} & = & {\vec{a}}_{N}+{\vec{a}}_{r}\\ {\vec{a}}_{N} & \equiv & -\vec{\nabla }\phi_{N}=-G\int \frac{\rho ({\vec{x}}^{\prime },t)}{R^{2}}\hat{R}d^{3}x^{\prime },\;\hat{R}\equiv \frac{\vec{R}}{R},\\ {\vec{a}}_{r} & \equiv & -\vec{\nabla }\phi_{r}=-\frac{G}{2c^{2}}\int \rho^{\left(2\right)}\left({\vec{x}}^{\prime },t\right)\hat{R}d^{3}x^{\prime } \end{array}$$

Now, let us examine a point particle with a mass $m_{j}$ positioned at ${\vec{r}}_{j}\left(t\right)$. The particle will possess a mass density given by

(11) $$\rho_{j}=m_{j}\delta^{\left(3\right)}\left({\vec{x}}^{\prime }-{\vec{r}}_{j}\left(t\right)\right)$$

Here, $\delta^{\left(3\right)}$ represents a three-dimensional Dirac delta distribution. This particle induces a Newtonian potential given by

(12) $$\phi_{Nj}=-G\frac{m_{j}}{R_{j}\left(t\right)},\;{\vec{R}}_{j}\left(t\right)=\vec{x}-{\vec{r}}_{j}\left(t\right),\;R_{j}\left(t\right)=\left|{\vec{R}}_{j}\left(t\right)\right|$$

and a retardation potential in the following form:

(13) $$\begin{array}{ccc} \phi_{rj} & = & -\frac{Gm_{j}}{2c^{2}}\frac{\partial^{2}}{\partial t^{2}}R_{j}\left(t\right)=\frac{Gm_{j}}{2c^{2}}\left({\hat{R}}_{j}\cdot {\vec{a}}_{j}-\frac{{\vec{v}}_{j}^{2}-{\left({\vec{v}}_{j}\cdot {\hat{R}}_{j}\right)}^{2}}{R_{j}\left(t\right)}\right),\\ \;{\hat{R}}_{j} & \equiv & \frac{{\vec{R}}_{j}}{R_{j}},\;{\vec{v}}_{j}\equiv \frac{d{\vec{r}}_{j}}{dt},\;{\vec{a}}_{j}\equiv \frac{d{\vec{v}}_{j}}{dt}. \end{array}$$

A test particle at the vicinity of particle *j* will be accelerated as follows:

(14) $$\begin{array}{ccc} {\vec{a}}_{Tj} & = & {\vec{a}}_{Nj}+{\vec{a}}_{rj}\\ {\vec{a}}_{Nj} & = & -\vec{\nabla }\phi_{Nj}=-G\frac{m_{j}}{R_{j}^{2}}{\hat{R}}_{j},\;{\vec{a}}_{rj}=-\vec{\nabla }\phi_{r}=\frac{Gm_{j}}{2R_{j}^{2}c^{2}}\left(R_{j}{\vec{a}}_{⊥j}+{\hat{R}}_{j}{\vec{v}}_{⊥j}^{2}-2\left({\vec{v}}_{j}\cdot {\hat{R}}_{j}\right){\vec{v}}_{⊥j}\right)\\ {\vec{a}}_{⊥j} & \equiv & {\vec{a}}_{j}-\left({\vec{a}}_{j}\cdot {\hat{R}}_{j}\right){\hat{R}}_{j},\;{\vec{v}}_{⊥j}\equiv {\vec{v}}_{j}-\left({\vec{v}}_{j}\cdot {\hat{R}}_{j}\right){\hat{R}}_{j}. \end{array}$$

in which the reader should not confuse the acceleration of the point particle *j* denoted ${\vec{a}}_{j}$ and the acceleration caused by particle on a test particle located at point $\vec{x}$ denoted ${\vec{a}}_{Tj}$.

## 4. The Inequality of Retarded Gravity

First, we notice that

(15) $$a_{Nj}=\left|{\vec{a}}_{Nj}\right|=G\frac{m_{j}}{R_{j}^{2}}\;\Rightarrow \;{\vec{a}}_{rj}=a_{Nj}\frac{\left(R_{j}{\vec{a}}_{⊥j}+{\hat{R}}_{j}{\vec{v}}_{⊥j}^{2}-2\left({\vec{v}}_{j}\cdot {\hat{R}}_{j}\right){\vec{v}}_{⊥j}\right)}{2c^{2}}$$

For nonrelativistic matter:

(16) $$\beta \equiv \frac{v}{c}\ll 1,$$

hence, we may approximately write:

(17) $${\vec{a}}_{rj}≃a_{Nj}\frac{R_{j}{\vec{a}}_{⊥j}}{2c^{2}},\;{\vec{a}}_{Tj}={\vec{a}}_{Nj}+{\vec{a}}_{rj}≃a_{Nj}\left(-{\hat{R}}_{j}+\frac{R_{j}{\vec{a}}_{⊥j}}{2c^{2}}\right).$$

Thus, in order for retarded gravity to have a significant effect, $\frac{R_{j}{\vec{a}}_{⊥j}}{2c^{2}}$ must be of the order of a prescribed fraction of unity fr ($|{\hat{R}}_{j}|=1$) or larger, in which fr=1%,10%,100% etc.; this leads to the retarded gravity inequality (to some readers this may be reminiscent of the quantum “uncertainty relation” while others may find the suggested resemblance shallow and even offensive, in any case this has nothing to do with the statistical nature of variables of quantum mechanics and their related standard deviations):

(18) $$\frac{R_{j}a_{⊥j}}{2c^{2}}>fr\;\Rightarrow \;R_{j}a_{⊥j}>2c^{2}fr\;\Rightarrow \;a_{⊥j}>a_{c}=\frac{2c^{2}fr}{R_{j}}.$$

Consider a point mass located on a circle that serves as a border of the M33 galaxy; then, we may ask what will be the amount of acceleration suffered by the point mass that will cause a retardation effect on a test particle located across the diameter of the galaxy (which is the furthest point on the imaginary circle from the point mass). Now, the radius of the galaxy M33 is Rs ≃ 30,000 light years = 2.8 × $10^{20}$ m. Hence, we will need an acceleration of about (we take fr=1):

(19) $$a_{c}=\frac{2c^{2}}{R_{j}}=\frac{2c^{2}}{2Rs}=\frac{c^{2}}{Rs}≃0.00032\;m/s^{2}$$

to observe the effect of retarded gravity. This does not seem to be such a huge acceleration and many point masses (atoms, molecules, etc.) in the galaxy may have accelerations that need to be considered in the total galactic balance of gravitational forces. From this point of view, we may partition the galactic point masses into two classes: Newtonian gravity particles and retarded (+Newtonian) gravity particles the difference depends on how big the Newtonian radius (which depends on the particle’s acceleration; we take fr=1 for simplicity, but the reader may choose another value of fr):

(20) $$\frac{R_{j}a_{⊥j}}{2c^{2}}<1\;R_{j}<R_{Nj}\equiv \frac{2c^{2}}{a_{⊥j}}.$$

**Figure 2:**
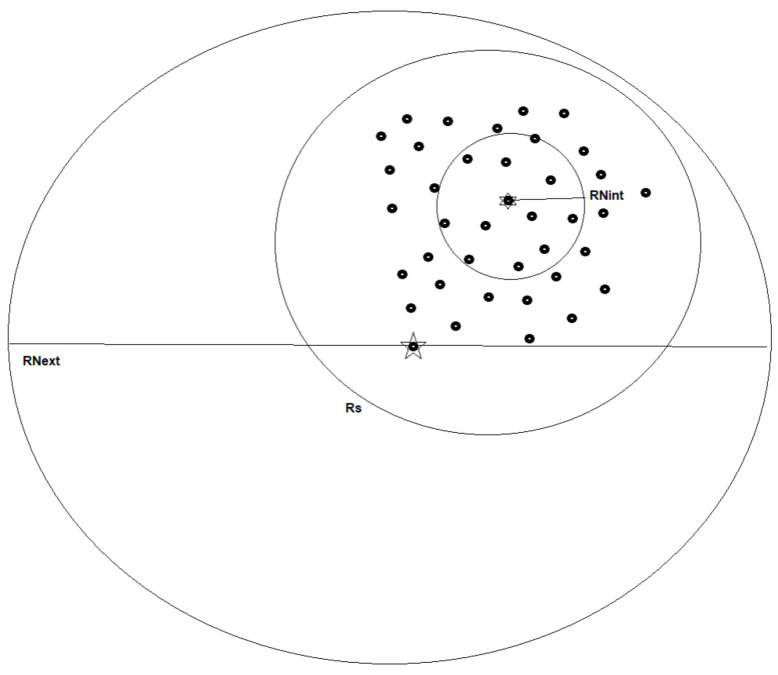
Point particles in the galaxy and their Newtonian spheres of influence. For some point particles, RNint is within the galaxy of Radius Rs; hence, retarded gravity due to those particles does affect the galaxy dynamics, while for other particles, RNext contains the entire galaxy, and hence those particle only cause gravitational Newtonian effects.

**Figure 3:**
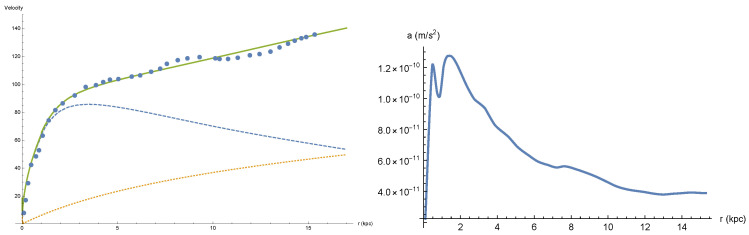
Rotation and acceleration curves for M33 [24]. On the left-hand side, we have the complete rotation curve, depicted by the solid line, representing the combined effect of two contributions: the dotted line represents the contribution from retardation, while the dashed line represents the contribution from Newtonian gravity [10]. On the right-hand side is the acceleration, assuming that the stars and gas move in circles around the galactic center and thus have an acceleration of $\frac{v^{2}}{r}$, where *r* denotes the distance from the galactic center.

This reality is depicted in Figure 2. Of course, each test particle is not affected by just one massive particle but by all $N_{p}$ massive particles this leads to the equation:

(21) $${\vec{a}}_{T}=\sum_{j=1}^{N_{p}}{\vec{a}}_{Tj}=\sum_{j=1}^{N_{p}}\left({\vec{a}}_{Nj}+{\vec{a}}_{rj}\right)=\sum_{j=1}^{N_{p}}{\vec{a}}_{Nj}+\sum_{j=1}^{N_{p}}{\vec{a}}_{rj}≃\sum_{j=1}^{N_{p}}{\vec{a}}_{Nj}+\sum_{j=1,j\in \mathrm{RG}}^{N_{p}}a_{Nj}\frac{R_{j}{\vec{a}}_{⊥j}}{2c^{2}}$$

in which RG means particles that have a retarded influence in point $\vec{x}$, that is, particles in which point $\vec{x}$ is outside their Newtonian radius. We point out that the stellar component of disk galaxies is not responsible for the retardation effects. To see this look at the velocity and acceleration curves of the M33 galaxy depicted in Figure 3:

Assuming stars (and gas) moving in circles, we obtain accelerations that do not exceed 1.4 × 10^−10^ m/s^2^, yielding a Newtonian radius not smaller than $R_{N}≃$ 4 × 10^7^ kpc; hence, the effects of those stars and rotating gas are completely Newtonian within the galaxy. Thus, to obtain retardation corrections, one must look at other processes taking place in a galaxy like stellar winds, supernovae explosions [9], and the depletion of gas in the intergalactic medium [10] leading to the deceleration of the rate of mass accretion by the galaxy and to an attractive gravitational retardation effect.

## 5. Conclusions

A recent paper [25] (see also [26,27,28]) highlights that measurements of gas speed in the outer regions of galaxies at high redshifts indicate a prevalence of steeply declining rotation curves, contrasting with the nearly universal flat rotation curves observed in nearby galaxies. This aligns with the proposition put forth by [10], suggesting that gas depletion plays a role in generating significant $\ddot{M}$ and implies that older galaxies should not exhibit significantly smaller $\ddot{M}$, resulting in steep rather than flat rotation curves. Furthermore, the paper suggests that once a smooth stellar disk forms within the baryonic matter, resembling properties of high-redshift galaxies, the computed rotation curves consistently remain relatively flat at large radii in the gas disk. Strikingly, only simulations devoid of a dark matter halo successfully replicate observed rotation curves. This finding supports our theory, which dismisses the presence of dark matter. Moreover, the paper implies that the flat rotation curves observed in low-redshift galaxies may either result from dark matter falling into the galactic potential well or necessitate an alternative explanation apart from dark matter. Indeed, an alternative explanation, considering retardation, is plausible.

This paper does not delve into dark matter on a cosmological scale, as that analysis is reserved for future work. Instead, it uses a retarded gravity approach that explains local dark matter phenomena, such as galactic rotation curves, gravitational lensing, and mass discrepancies inferred from the virial theorem. However, retarded gravity, which is based on weak-field approximations and a Lorentzian metric, is not suitable for cosmological scales without further adaptation. An early attempt [29] was made to correct for these scales through perturbations in the Friedman–Robertson–Walker metric. This may eventually explain phenomena like the cosmic microwave background (CMB) spectrum and galaxy formation. A complete cosmological model would also need to explain dark energy in addition to dark matter to derive the results of ΛCDM cosmology.

Recently, new approaches to cosmology were suggested by Gupta [30,31,32], with a model in which the speed of light and gravitational constant vary over time. This leads to modified Friedman–Robertson–Walker equations that produce dark-energy-like effects without invoking dark energy and predict an older universe, potentially explaining distant, mature galaxies observed by the James Webb Space Telescope. This approach complements the current approach, which is only applicable up to galaxy cluster scales.

Additionally, recently, ref. [33] proposed an initial Euclidean phase of the universe, avoiding an initial singularity and allowing for early rapid processes that could account for mature galaxies at high redshifts but without postulating temporal changes in nature’s fundamental constants. This model, as well as the retarded gravity approach, may offer complementary explanations for dark matter on different scales, with future perturbation analysis of the hybrid Euclidean–Lorentzian model possibly shedding further light on these issues. 

## Data Availability

The original contributions presented in the study are included in the article, further inquiries can be directed to the corresponding author.
